# Machine Learning Models for Predicting Stroke Mortality in Malaysia: An Application and Comparative Analysis

**DOI:** 10.7759/cureus.50426

**Published:** 2023-12-13

**Authors:** Che Muhammad Nur Hidayat Che Nawi, Suhaily Mohd Hairon, Wan Nur Nafisah Wan Yahya, Wan Asyraf Wan Zaidi, Kamarul Imran Musa

**Affiliations:** 1 Department of Community Medicine, School of Medical Sciences, Universiti Sains Malaysia, Kubang Kerian, MYS; 2 Department of Internal Medicine, Universiti Kebangsaan Malaysia Medical Centre (UKMMC), Kuala Lumpur, MYS; 3 Department of Community Medicine, School of Medical Sciences, Universiti Sains Malaysia, Kota Bharu, MYS

**Keywords:** malaysia, stroke mortality, comparative analysis, prediction models, machine learning

## Abstract

Background

Stroke is a significant public health concern characterized by increasing mortality and morbidity. Accurate long-term outcome prediction for acute stroke patients, particularly stroke mortality, is vital for clinical decision-making and prognostic management. This study aimed to develop and compare various prognostic models for stroke mortality prediction.

Methods

In a retrospective cohort study from January 2016 to December 2021, we collected data from patients diagnosed with acute stroke from five selected hospitals. Data contained variables on demographics, comorbidities, and interventions retrieved from medical records. The cohort comprised 950 patients with 20 features. Outcomes (censored vs. death) were determined by linking data with the Malaysian National Mortality Registry. We employed three common survival modeling approaches, the Cox proportional hazard regression (Cox), support vector machine (SVM), and random survival forest (RSF), while enhancing the Cox model with Elastic Net (Cox-EN) for feature selection. Models were compared using the concordance index (C-index), time-dependent area under the curve (AUC), and discrimination index (D-index), with calibration assessed by the Brier score.

Results

The support vector machine (SVM) model excelled among the four, with three-month, one-year, and three-year time-dependent AUC values of 0.842, 0.846, and 0.791; a D-index of 5.31 (95% CI: 3.86, 7.30); and a C-index of 0.803 (95% CI: 0.758, 0.847). All models exhibited robust calibration, with three-month, one-year, and three-year Brier scores ranging from 0.103 to 0.220, all below 0.25.

Conclusion

The support vector machine (SVM) model demonstrated superior discriminative performance, suggesting its efficacy in developing prognostic models for stroke mortality. This study enhances stroke mortality prediction and supports clinical decision-making, emphasizing the utility of the support vector machine method.

## Introduction

In the last 30 years, the challenge posed by stroke has been constant, with rising death and disability rates [1]. This reality underscores the need for improved methods that support treatment choices and the management of prognostic outlooks. A critical strategy is the prediction of long-term outcomes, such as stroke mortality, in patients with acute stroke. Prediction models, which integrate patient data and healthcare procedures to predict specific future health events (prognostication), especially stroke mortality, are crucial in preventing strokes [2].

Recently, models like the Framingham score, cardiovascular disease risk algorithm (QRISK), Reynolds risk scores, and the European System for Cardiac Operative Risk Evaluation (EUROSCORE) have been applied to stroke, serving functions ranging from healthcare planning to clinical trial selection [3-6]. However, these models are founded on traditional regression methods such as binary logistic regression and Cox proportional hazards regression (Cox).

Predictive models derived from binary logistic regression do not account for the time-to-event factor, and Cox regression is constrained by strict proportional hazard assumptions. Moreover, they assume that continuous covariates exert a linear influence on the logarithm of the hazard, which real-world data may not always adhere to [7]. In contrast, machine learning methods, compared to Cox regression, sidestep parametric or semi-parametric assumptions and demonstrate the ability to detect and accommodate higher-order interactions and nonlinear relationships [8].

While machine learning prediction models for stroke mortality exhibit commendable accuracy [2], concerns have emerged regarding their practical utility and clinical application, particularly when these models lack validation, comprehensive reporting, and accessibility for clinical use [9].

The Cox proportional hazards regression is a staple in survival analysis, estimating the likelihood of an event over time based on predictor variables [10]. Elastic net (EN) is a feature selection method combining the least absolute shrinkage and selection operator (LASSO) and ridge regression techniques to overcome their respective limitations [11]. While ridge regression includes all variables, LASSO might ignore correlated predictors. EN tends to select multiple correlated variables together.

Support vector machine (SVM) is a versatile machine learning algorithm for classification and regression [12]. It represents classes in a multidimensional space, seeking a hyperplane that divides the dataset with the largest margin, minimizing errors. SVM adaptations for survival analysis handle censored data through regression or by combining regression and ranking, although the latter can be impractical for larger datasets because of its computational intensity.

Random survival forest (RSF) is an ensemble method tailored for survival analysis of right-censored data, expanding the random forest technique [13]. RSF grows trees using bootstrap samples, selecting features, and splitting nodes based on survival criteria that include both survival time and censoring.

In our study, we developed both traditional and machine learning-based prognostic prediction models for stroke patients treated in five major hospitals in Peninsular Malaysia. Our objective is to compare the performance of these different models to select the optimal predictive model, offering insights into the advancement of machine learning in predicting stroke mortality using data from acute stroke patients diagnosed between January 1, 2016, and December 31, 2021.

## Materials and methods

Study design

This retrospective cohort study encompassed patients diagnosed with stroke between January 1, 2016, and December 31, 2021, in five hospitals located in Peninsular Malaysia. Patient data, including demographics, comorbidities, and intervention records, were collected from the medical records of these hospitals. Information on patient outcomes (death) was obtained through a data linkage process with the Malaysian National Mortality Registry's death records. If there is no data on death until December 31, 2021, then such patients are considered censored (administrative censors) [14].

Study location

To ensure that our study represents the diverse Malaysian population in Peninsular Malaysia, we meticulously chose five healthcare centers: Hospital Universiti Sains Malaysia (HUSM), Hospital Canselor Tuanku Muhriz Universiti Kebangsaan Malaysia (HCTM UKM), Hospital Seberang Jaya (HSJ), Hospital Raja Perempuan Zainab II (HRPZ II), and Hospital Sultanah Nur Zahirah (HSNZ). These centers are equipped with established stroke units staffed by a team of healthcare professionals, including neurologists, neurosurgeons, and neuroradiologists. These units are crucial referral hubs within their respective geographical regions. We obtained ethical clearance to conduct our research within these hospitals.

Study duration

The study commenced in September 2022 and concluded in September 2023 spanning a 12-month period. This timeline included securing ethical clearance over the initial two months, followed by a four-month data collection phase until February 2023. Subsequently, data analysis and report writing continued until September 2023.

Study population

This study's reference population consisted of acute stroke patients diagnosed in Kuala Lumpur, Penang, Terengganu, and Kelantan. Conversely, the source population comprised acute stroke patients diagnosed and receiving medical care at the aforementioned hospitals, with incident dates falling within January 1, 2016, to December 31, 2021.

The sampling frame comprised acute stroke patients who received care at these hospitals, were aged 18 years and older, held Malaysian nationality, and had a primary diagnosis of stroke. Patients with underlying shock, hypertensive encephalopathy, or a diagnosis of transient ischemic attack (TIA) were excluded.

As of March 1, 2023, comprehensive medical data and follow-up information regarding vital status were meticulously collected from a cohort of 987 patients. After excluding foreign cases (n = 21) and removing duplicate cases (n = 16), the final dataset for analysis included 950 patients with eighteen variables.

Data preparation

Our data preparation commenced with an assessment of data quality and consistency across all five study sites. It is worth emphasizing that we had previously established a study proforma and diligently trained our data enumerators. This meticulous approach ensured that our dataset remained free from quality issues, such as instances when data types were mixed (e.g., age recorded both numerically and categorically) or conflicting data values (e.g., "woman" and "female") arose. Furthermore, we conducted an outlier analysis.

In the subsequent step, we effectively managed missing values through single imputation by chained equations [15,16]. Single imputation involves creating imputed datasets utilizing various models tailored to features with missing data. It is important to note that, in this study, we opted for a single imputation, meaning that each missing value was replaced by a single imputed value based on predictive models. Further details concerning missing values for different candidate features are available in Appendix 1.

Statistical analysis

In our statistical analysis, we aimed to predict stroke mortality using three distinct survival modeling approaches: Cox proportional hazard regression (Cox), support vector machine (SVM), and RSF, while employing EN for feature selection [10,12,13].

We conducted the statistical analysis of data from acute stroke patients across five study sites using a combination of Python version 3.12.0 (Python Software Foundation, Wilmington, DE) and R software version 4.3.1 (R Foundation for Statistical Computing, Vienna, Austria) [17], leveraging libraries such as pandas, NumPy, eli5, and scikit-survival [18-20].

In this study, Python was primarily employed for various analyses, including data preprocessing, model training and evaluation, and generating various statistical metrics such as the area under the curve (AUC). Specifically, Python was used to develop and assess machine learning models for predicting stroke mortality.

On the other hand, R was utilized for specific tasks, such as in comparing the C-index of different models and generating receiver operating characteristic (ROC) curves. Additionally, R was used to output the predictive scores required for these comparisons. This combined usage of Python and R allowed us to leverage the strengths of each programming language for different aspects of our analysis.

The dataset was initially divided into training and testing sets, with model development conducted on the training data, and model evaluation performed on testing data. To ensure that the model generalizes well across all classes, we took steps to address the issue of class imbalance within the dataset.

To mitigate the effects of class imbalance, we employed appropriate techniques during the training phase. These techniques may include oversampling the minority class, undersampling the majority class, or using a combination of both. Additionally, we applied stratified sampling to maintain the class distribution in both the training and testing sets, preserving the relative proportions of different classes.

By addressing the imbalance issue, we aimed to enhance the model's ability to generalize and make accurate predictions for all classes within the dataset.

In our study, we employed a conventional feature selection approach specifically for the Cox model. This approach involved an initial univariate Cox model to identify features that were both statistically significant (P<0.1) and clinically relevant. These selected features were then integrated into a multivariate Cox regression model.

It is important to note that feature selection was performed exclusively for the Cox model in our analysis. Other machine learning models used in this study, such as the SVM and any other models, were not subjected to the same feature selection process. This distinction ensured that predictors with direct associations and clinical relevance to the Cox model were identified while allowing other models to consider the full set of predictors without restrictions.

Traditionally, predictors in the Cox model should adhere to the proportional hazard assumption. However, the primary aim of our modeling in this study was survival prediction, prioritizing the maximization of the concordance index (C-index) and time-dependent AUC. Consequently, we did not conduct the test for proportional hazards during the modeling process [21].

In the Cox model with EN (Cox-EN) analysis, we examined the influence of various alpha values on model coefficients and conducted cross-validation to identify the optimal alpha and feature subset. Alpha in the Cox-EN model represents a hyperparameter that controls the balance between L1 (Lasso) and L2 (Ridge) regularization penalties. A higher alpha value emphasizes L1 regularization, which encourages sparsity by shrinking some coefficients to exactly zero, effectively selecting a subset of important features.

Through cross-validation, we determined the optimal alpha value that yielded the best model performance and feature selection. This selected alpha value was then used to fit the Cox-EN model, and we assessed feature importance. To visually present the significance of individual features in the model's predictions, we created a horizontal bar plot.

For the RSF analysis, we conducted hyperparameter tuning using a randomized search approach to optimize the model's performance. The tuning process aimed to find the best combination of hyperparameters for the RSF model. Importantly, this tuning process did not utilize the validation data in any way to prevent data leakage and ensure unbiased model evaluation. Additionally, we fine-tuned the SVM model through hyperparameter adjustments and cross-validation to enhance its predictive accuracy. Further details regarding the hyperparameter tuning process for the RSF model and SVM model development and optimization are available in Appendixes 3 and 4, respectively. 

Evaluation of model performance

We assessed the discriminative performance of our models using three metrics: the C-index, time-dependent AUC, and the D-index [22-24]. The C-index and time-dependent ROC curves were computed using Hmisc, survcomp, and survival ROC packages in R software [25-27]. The C-index provides an overall measure of a model's discriminative capability, while the time-dependent AUC evaluates how well the model's predicted probabilities align with the actual binary survival status, even when considering censored observations at specific time points. Both the C-index and time-dependent AUC produce values within the ranges of 0 to 1, where 0.5 signifies performance equivalent to random guessing and 1 indicates perfect discrimination.

To further gauge the discriminative power of our models, we employed the D-index, a metric that measures the degree of separation between patients in equally sized high-risk and low-risk groups. These categorizations were based on risk scores obtained from different models. Higher D-index values signify a more pronounced discriminative ability of the model. Further explanation regarding the categorization of high- and low-risk groups was provided in Appendix 5. 

Survival curves for high-risk and low-risk groups were estimated using the Kaplan-Meier method. The categorization into high-risk and low-risk groups was based on a threshold applied to the risk scores obtained from our models. Specifically, patients with risk scores exceeding the defined threshold were categorized as high-risk, while those below the threshold were categorized as low-risk.

The log-rank test was employed to compare these survival curves and assess the statistical significance of the differences in survival between the two groups. The log-rank test provides a test statistic that quantifies whether there is a statistically significant difference in survival outcomes between the high-risk and low-risk groups.

Additionally, we evaluated the calibration performance of our models using the Brier score, which ranges from 0 to 1 [28]. It is important to note that the interpretation of the Brier score's values can vary depending on the dataset's outcome prevalence. For datasets with balanced outcomes, a lower Brier score indicates better-calibrated predictions, with 0.25 considered comparable to random guessing. However, when dealing with datasets where the outcome is less prevalent, random guessing may result in a Brier score lower than 0.25. The specific threshold for a well-calibrated model in our dataset, given its outcome prevalence, will be reported in the results section to provide a context-specific interpretation of the Brier score.

**Figure 1:**
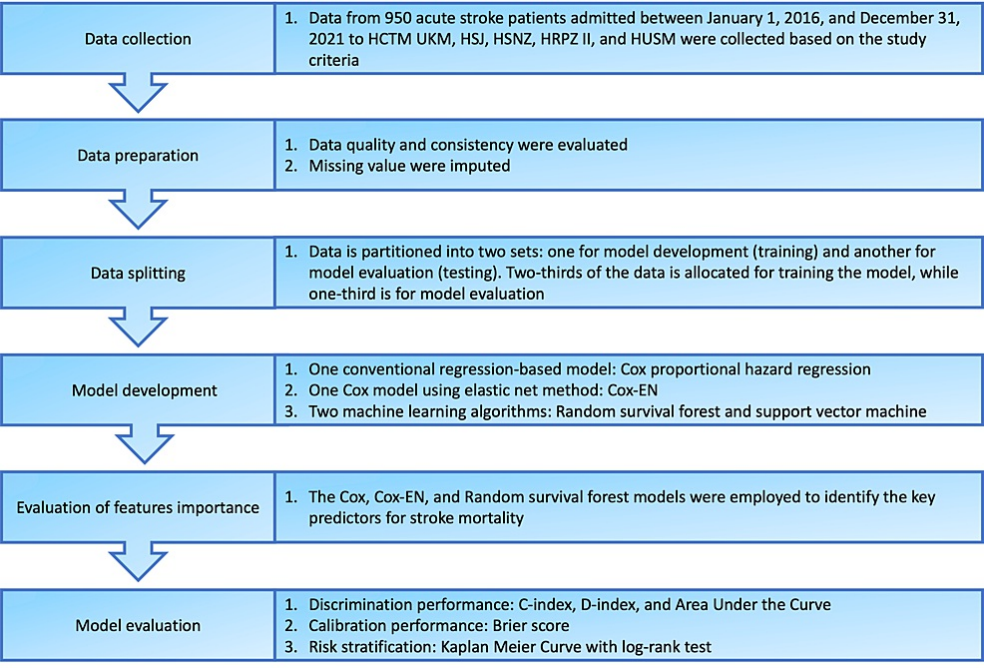
Graphical illustration of the study workflow HCTM UKM: Hospital Canselor Tuanku Muhriz Universiti Kebangsaan Malaysia; HSJ: Hospital Seberang Jaya; HSNZ: Hospital Sultanah Nur Zahirah; HRPZ II: Hospital Raja Perempuan Zainab II; HUSM: Hospital Universiti Sains Malaysia; Cox: Cox proportional hazard regression; Cox-EN: Cox model with elastic net; C-index: Concordance index; D-index: Discrimination index.

The metrics performance was computed at different time points: three months, one year, and three years. A visual representation delineating the procedural framework employed in this study is depicted in Figure 1.

Ethical approval

This study adhered to the principles outlined in the Declaration of Helsinki. Ethical approval was obtained from the Human Research and Ethics Committee, Universiti Sains Malaysia, USM/JEPeM/22060451, and the Medical Review and Ethical Committee from the National Institute of Health, Ministry of Health Malaysia, NMRR ID-22-01570-UQ4 (IIR).

## Results

Participants’ characteristics and model statistics

**Figure 2:**
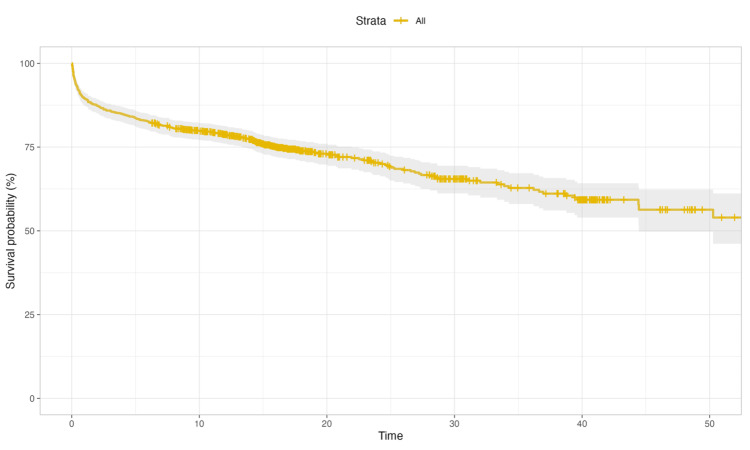
Overall survival of acute stroke patients

In this study, a cohort of 950 stroke patients was enrolled, and, among them, 272 patients experienced mortality during the follow-up period. The median survival time for the entire patient cohort remained undefined because less than 50% of patients had reached the midpoint of the survival data (Figure 2). The trend appears to be a gradual decline in survival probability as time progresses. Initially, there is a steeper drop, indicating a higher rate of mortality shortly after the stroke event. As time continues, the rate of decline in survival probability slows down but continues to decrease steadily.

To predict mortality among stroke patients, we employed four distinct prognostic models: Cox, Cox-EN, SVM, and RSF. These models were trained using a designated dataset, and their performance was subsequently assessed using independent test data. We evaluated their predictive accuracy through the following metrics: C-index, time-dependent AUC, D-index, and Brier score.

All the models demonstrated strong calibration, indicating their reliability. Among them, the SVM model exhibited a C-index of 0.803 (95% CI: 0.758, 0.847).

Evaluation of feature importance

**Figure 3:**
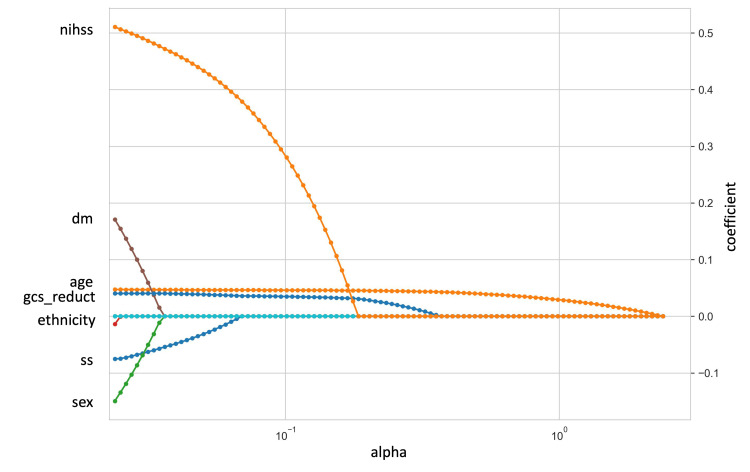
The coefficient of features change for varying alphas for the Cox-EN model nihss: National Institutes of Health Stroke Scale; dm: Diabetic status; age: Age in years; gcs_reduct: Glasgow Coma Scale reduction; ethnicity: Ethnicity; ss: Study site; sex: Gender of the patients.

**Figure 4:**
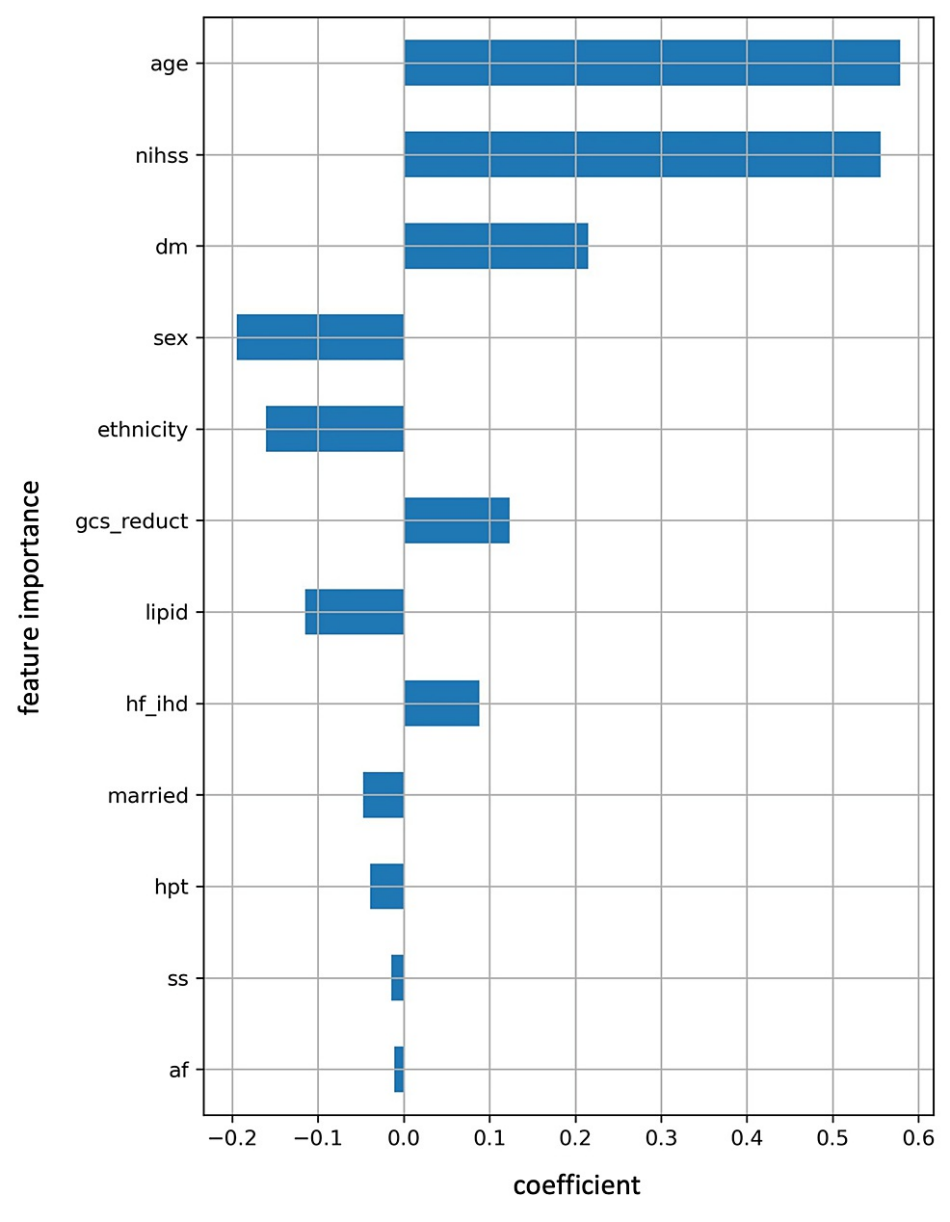
The important coefficient of each feature corresponding to the optimal α by an elastic net age: Age in years; nihss: National Institutes of Health Stroke Scale; dm: Diabetic status; sex: Gender of the patients; ethnicity: Ethnicity; gcs_reduct: Glasgow Coma Scale reduction; lipid: Hyperlipidemia; hf_ihd: Heart disease; married: Marital status; hpt: Hypertension status; ss: Study site; af: Atrial fibrillation.

To identify the key predictors of stroke mortality, we initially employed EN feature selection, resulting in the identification of 12 significant features. Figure 3 illustrates the variation in coefficients across different alpha values, while Figure 4 presents the coefficients of each feature corresponding to the optimal alpha value. The top five critical features, ranked by their contribution, were age, National Institutes of Health Stroke Scale (NIHSS), diabetes status, Glasgow Coma Scale (GCS) reduction, and heart disease. All of these features were positively associated with a higher risk of stroke mortality.

**Figure 5:**
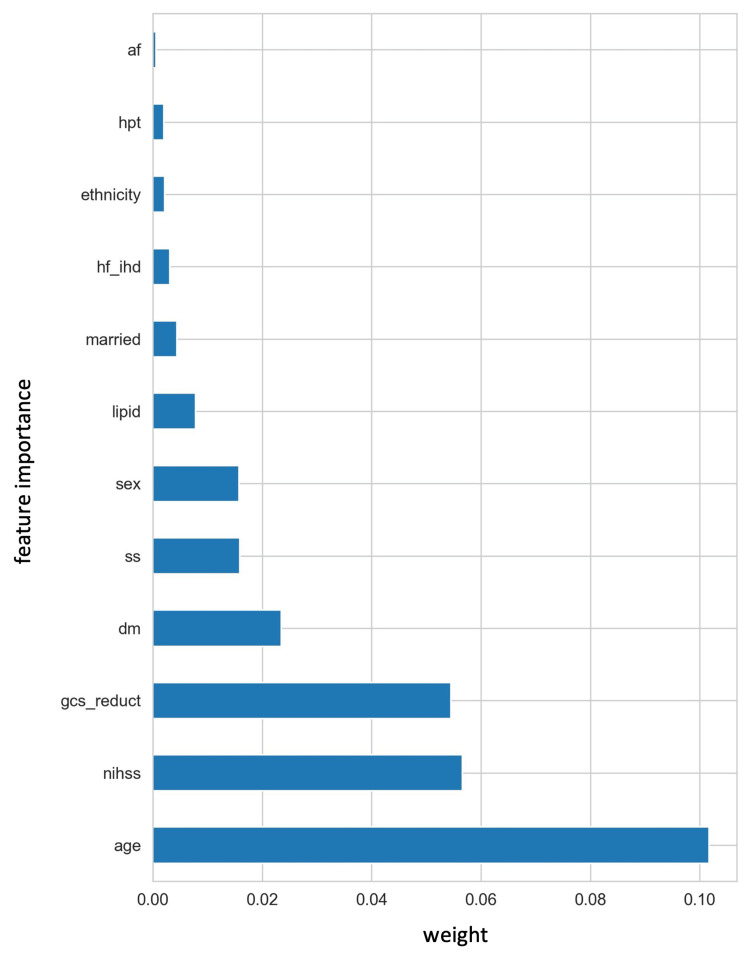
The important coefficient of each feature by random survival forest af: Atrial fibrillation; hpt: Hypertension status; ethnicity: Ethnicity; hf_ihd: Heart disease; married: Marital status; lipid: Hyperlipidemia; sex: Gender of the patients; ss: Study site; dm: Diabetic status; gcs_reduct: Glasgow Coma Scale reduction; nihss: National Institutes of Health Stroke Scale; age: Age in years.

Subsequently, RSF was employed to rank the important features selected by EN. In our RSF analysis, feature importance was assessed using permutation importance, a technique that measures the impact of individual features on the model's predictive performance. Figure 5 displays the results, with the top five influential features identified as age, National Institutes of Health Stroke Scale (NIHSS), Glasgow Coma Scale (GCS) reduction, diabetes status, and study sites.

In Appendix 2, we present the outcomes of univariate and multivariate Cox analyses, respectively. The features found to be statistically significant in the univariate Cox analysis included age, GCS score, gender, diabetes mellitus, chronic kidney disease, atrial fibrillation, heart disease, hyperlipidemia, smoking status, NIHSS score, and intravenous thrombolysis. Upon further exploration, we identified seven significant features associated with mortality among stroke patients in Malaysia, namely, age, diabetes mellitus, hyperlipidemia, GCS score, NIHSS score, and intravascular thrombolysis.

Models’ performance

The evaluation results for the four models are presented in Table 1. In terms of the C-index, the SVM model demonstrated a slight superiority in discriminative ability compared to the Cox model (0.796; 95% CI: 0.752, 0.839), the Cox-EN model (0.796; 95% CI: 0.752, 0.839), and the RSF model (0.794; 95% CI: 0.749, 0.843).

**Table 1:** Performance of different machine learning methods

Indexes	Cox	Cox-EN^a^	SVM^b^	RSF^c^
C-index^d^ (95% CI)	0.796 (0.752, 0.839)	0.796 (0.752, 0.839)	0.803 (0.758, 0.847)	0.794 (0.749, 0.843)
AUC^e ^(three months)	0.834	0.834	0.842	0.836
AUC (one year)	0.845	0.845	0.846	0.835
AUC (three years)	0.783	0.783	0.791	0.782
D-index (95% CI)	5.18 (3.73, 7.18)	5.18 (3.73, 7.18)	5.31 (3.86, 7.30)	6.79 (4.62, 9.98)
Brier score (three months)	0.1102	0.1102	0.1102	0.1015
Brier score (one year)	0.1326	0.1326	0.1326	0.1339
Brier score (three years)	0.2203	0.2203	0.2203	0.2154
^a^EN: elastic net, ^b^SVM: support vector machine, ^c^RSF: random survival forest, ^d^C-index: concordance index, ^e^AUC: area under the curve				

**Figure 6:**
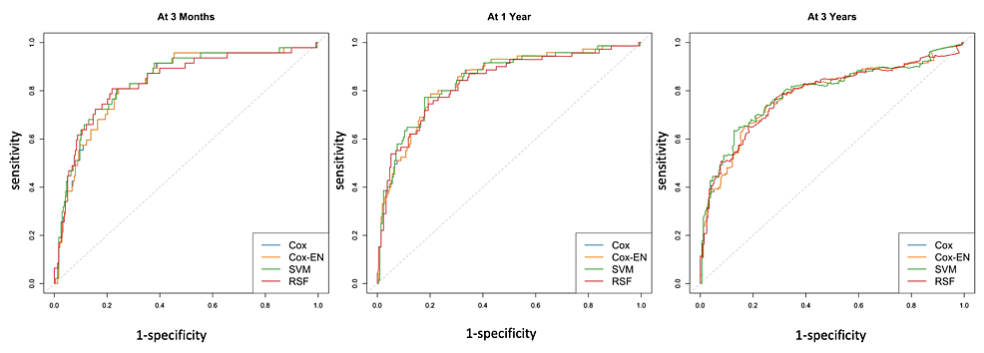
Time-dependent receiver operating characteristic curves of models at three months, one year, and three years Cox: Cox proportional hazard regression; Cox-EN: Cox model with elastic net; RSF: Random survival forest; SVM: Support vector machine.

**Figure 7:**
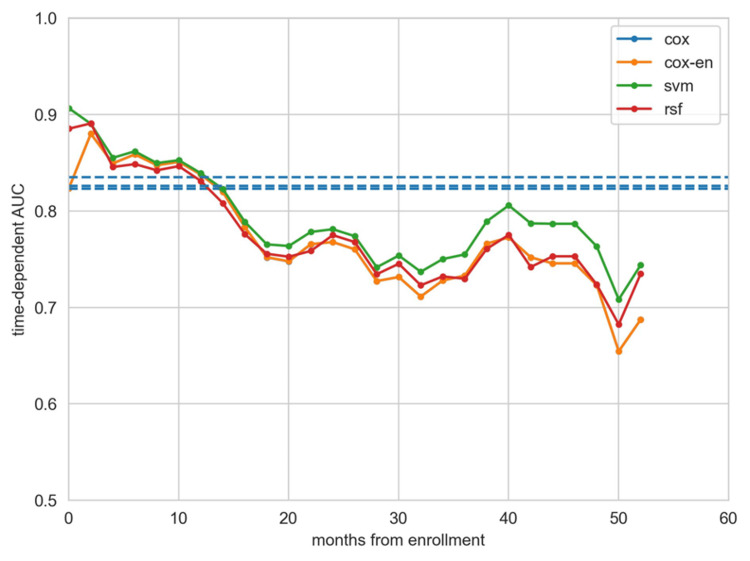
Time-dependent AUC of models over time AUC: Area under the curve; Cox: Cox proportional hazard regression; Cox-EN: Cox model with elastic net; RSF: Random survival forest; SVM: Support vector machine. The blue dotted line represents the mean area under the curve.

Figure 6 displays the time-dependent receiver operating characteristic curves for each model at intervals of one month, one year, and three years. Meanwhile, Figure 7 illustrates the time-independent AUC for each model over time, with SVM consistently exhibiting the highest AUC across most time points.

**Figure 8:**
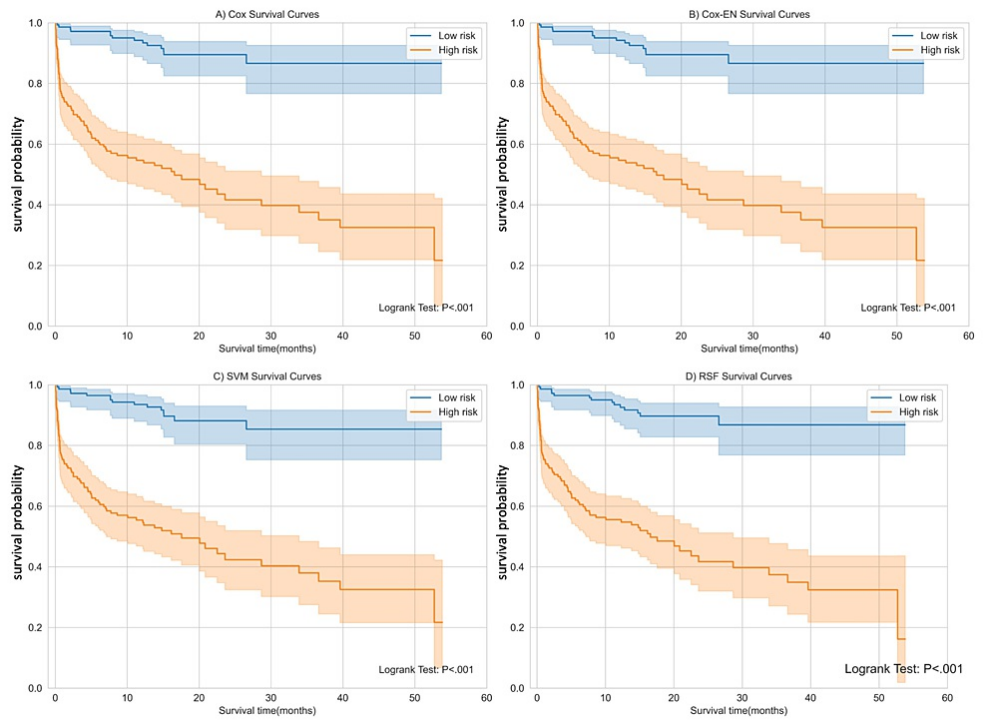
Survival curves of high-risk and low-risk groups divided according to the risk score from (A) Cox: Cox proportional hazard regression, (B) Cox-EN: Cox model with elastic net, (C) SVM: support vector machine, and (D) RSF: random survival forest

To assess the risk stratification ability of the models, we present the survival curves for high-risk and low-risk groups based on the risk score in Figure 8. Regarding the D-index, the SVM model (5.31; 95% CI: 3.86, 7.3) ranked second, following the RSF model (6.79; 95% CI: 4.62, 9.98). This implies that patients in the high-risk group have a 5.31 times higher risk of mortality than those in the low-risk group.

Notably, all four models exhibited Brier scores below 0.25 for one-month, one-year, and three-year predictions, indicating strong calibration and confirming their reliability.

## Discussion

The primary objective of this investigation was the development and assessment of machine learning prognostic models designed to predict stroke mortality in a sample of 950 patients from Peninsular Malaysia. Our study yielded several critical findings carrying significant clinical implications. Notably, the median survival time for the entire patient cohort remained indeterminate. This emphasizes the need for more extended follow-up periods. In our exploration of machine learning models, the support vector machine (SVM) model emerged as the most capable, demonstrating superior discriminative performance with a C-index of 0.803 (95% CI: 0.758, 0.847). Furthermore, the application of feature selection techniques, including EN, RSF, and cox proportional hazard regression, consistently identified four pivotal features: age, diabetes status, GCS reduction, and the NIHSS. These shared key features play a pivotal role in predicting stroke mortality, further underscoring their clinical relevance and potential for enhancing patient outcomes.

The undefined median survival time observed in our study, primarily because of the relatively short follow-up duration with less than 50% of patients reaching the midpoint of survival data, underscores the challenges of obtaining comprehensive long-term data in stroke mortality research [29]. This highlights the need for extended follow-up periods to capture the full spectrum of patient outcomes in the context of stroke's multifaceted nature [30]. However, this observation also emphasizes the practical limitation of protracted follow-up durations in clinical settings. Thus, it accentuates the critical requirement for predictive models, particularly machine learning-based approaches, capable of providing early insights into patient outcomes [9].

All four models, including Cox, Cox-EN, SVM, and RSF, exhibited robust calibration, affirming their reliability for clinical applications. Particularly noteworthy was the SVM model, which displayed superior discriminative prowess, boasting a C-index of 0.803 (95% CI: 0.758, 0.847). Our findings align with previous research outcomes, as evidenced by a local study comparing various machine learning algorithms within a single-center cohort, wherein SVM demonstrated commendable performance comparable to RSF in predicting stroke mortality (C-index: 0.920; 95% CI: 0.84, 0.97) [31]. Additionally, in a retrospective cohort investigation comparing multiple machine learning algorithms, SVM (C-index: 0.865; 95% CI: 0.81, 0.92) outperformed other classifiers, such as decision trees and RSF, in predicting stroke mortality [32]. These findings underscore the promise of machine learning techniques in enhancing the precision of stroke mortality prediction. Notably, the SVM model's superior C-index signifies its effectiveness in distinguishing between high-risk and low-risk patient groups. This suggests the practical potential of SVM in optimizing patient risk stratification and, consequently, healthcare interventions.

The consistent identification of four crucial features through our feature selection methods, namely, age, diabetes status, GCS reduction, and NIHSS, carries substantial clinical implications. These features have proven to be pivotal in forecasting stroke mortality, underscoring their critical role in risk assessment and patient management. Age, for instance, is associated with an escalating incidence of stroke [33], translating into elevated risks of mortality, poorer functional outcomes, extended hospitalization, and the need for institutional care. Notably, stroke fatality exhibits an increase from 8.6% to as high as 24.2% as age progresses from 60 to over 80 years [34]. Additionally, individuals with diabetes face a 23% higher risk of stroke mortality when compared to those without diabetes [35]. Moreover, the incorporation of clinical assessment tools such as GCS and NIHSS emerges as indispensable in evaluating the neurological and functional statuses of stroke patients. These assessment scales provide invaluable insights into the stroke's severity and its impact, empowering healthcare professionals to customize interventions and treatment approaches [36-38]. The identification of GCS and NIHSS as key features underscores the profound importance of conducting comprehensive neurological assessments in the context of predicting stroke mortality.

Our study boasts several strengths, including a substantial sample size of 950 stroke patients and rigorous methodology. The comprehensive analysis employing machine learning techniques offers advantages over traditional methods. This allows for the detection of higher-order interactions and nonlinear relationships, which can enhance predictive accuracy. The presence of strong calibration across all models indicates their reliability in a clinical context, which is a substantial strength of our study. However, our research is not without limitations. Data with missing values were imputed using single imputation by chained equation, which, while robust, is not without its uncertainties. The study population was confined to in-hospital patients, excluding those in out-of-hospital settings, which may introduce a selection bias. The retrospective cohort study design inherently carries biases that must be considered when interpreting the results. Furthermore, incomplete follow-up for some patients necessitates caution when generalizing our findings. It is essential to acknowledge the inherent challenges posed by real-world clinical data, which include potential biases and confounding variables. Finally, while machine learning models demonstrate high predictive accuracy, their practical utility in clinical settings may be compromised if they lack full validation and are not readily available for clinical use.

## Conclusions

In conclusion, our study significantly advances the field of stroke mortality prediction by comparing multiple prognostic models. We highlight the critical features, including age, diabetic status, GCS reduction, and NIHSS, which play a pivotal role in predicting stroke outcomes. While the SVM model demonstrated exceptional discriminative ability, it is important to note that other models in our analysis performed remarkably well, showcasing the robustness of the approaches employed. Our research offers valuable insights for healthcare practitioners and serves as a foundation for further research aimed at enhancing patient care and prognostic expectations management in stroke cases.

## Figures and Tables

**Figure 9:**
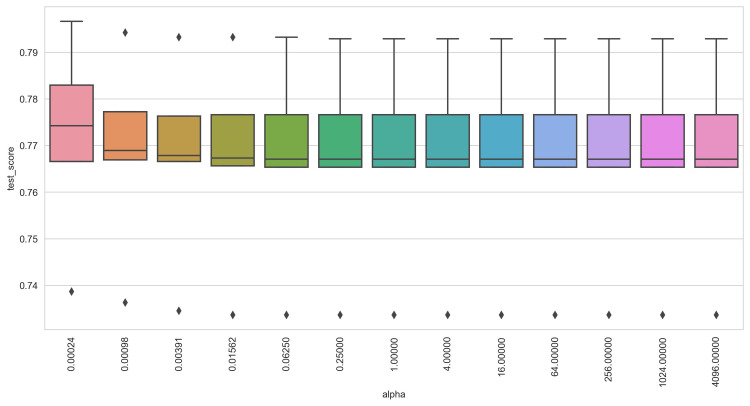
Performance of the SVM model across different alpha values during hyperparameter tuning

## Appendices

Appendix 1

**Table 2:** Missing data percentage for each feature

Features’ initials	Features’ name	Total	Percent missing
age	Age	956	0.9
sex	Sex	956	0.1
ethnicity	Ethnicity	956	0.1
married	Marital status	956	34.2
dm	Diabetes mellitus	956	2.3
hpt	Hypertension	956	1.4
ckd	Chronic kidney disease	956	15.8
af	Atrial fibrillation	956	3.7
hf_ihd	Heart disease	956	15.7
lipid	Hyperlipidaemia	956	3.0
smoke	Smoking status	956	8.2
who	Diagnosis	956	0.2
gcs_reduct	GCS reduction	956	6.2
nihss	NIHSS	956	11.5
iv_thrombolysis	Thrombolysis	956	6.2
iv_thrombectomy	Thrombectomy	956	6.2
status	Status on follow-up	956	0

Appendix 2

**Table 3:** Results for the simple and multiple Cox proportional hazard regression models

Variables	N	HR^1^	95% CI^1^	P value	HR^2^	95% CI^2^	P value
Age	950	1.05	1.04, 1.06	<0.001	1.04	1.03, 1.05	<0.001
GCS score	950	1.08	1.06, 1.11	<0.001	0.95	0.91, 0.99	0.015
Gender	950						
Female		—	—		—	—	
Male		0.64	0.50, 0.81	<0.001	0.88	0.68, 1.13	0.3
Ethnicity	950						
Malay		—	—				
Chinese		0.87	0.58, 1.28	0.5			
Indian		0.48	0.21, 1.07	0.073			
Others		0.00	0.00, Inf	>0.9			
Marital status	950						
Married		—	—				
Single		0.74	0.42, 1.30	0.3			
Others		0.96	0.67, 1.38	0.8			
Diabetes mellitus, Yes	950						
No		—	—				
Yes		1.41	1.11, 1.79	0.005			
Hypertension, Yes	950						
No		—	—				
Yes		1.26	0.91, 1.74	0.2			
Chronic kidney disease, Yes	950						
No		—	—				
Yes		1.59	1.06, 2.40	0.027			
Atrial fibrillation, Yes	950						
No		—	—				
Yes		1.84	1.25, 2.73	0.002			
Heart disease, Yes	950						
No		—	—				
Yes		1.42	1.03, 1.97	0.034			
Hyperlipidemia, Yes	950						
No		—	—		—	—	
Yes		0.75	0.59, 0.96	0.021	0.52	0.40, 0.69	<0.001
Smoking status, Yes	950						
No		—	—				
Yes		0.67	0.48, 0.94	0.019			
Diagnosis (WHO)	950						
Ischemic stroke		—	—				
Nonischemic stroke		1.46	0.98, 2.18	0.061			
NIH stroke scale (NIHSS)	950						
No stroke symptoms (0)		—	—		—	—	
Minor stroke (1-4)		0.49	0.27, 0.88	0.017	1.12	0.59, 2.13	0.7
Moderate stroke (5-15)		1.32	0.77, 2.27	0.3	2.06	1.14, 3.70	0.016
Moderate to severe stroke (16-20)		2.26	1.27, 4.03	0.006	2.36	1.20, 4.65	0.013
Severe stroke (21-42)		4.25	2.41, 7.49	<0.001	4.95	2.58, 9.53	<0.001
Intervention (thrombolysis), Yes	950						
No		—	—		—	—	
Yes		1.51	1.04, 2.18	0.029	0.42	0.27, 0.67	<0.001
Intervention (thrombectomy), Yes	950						
No		—	—				
Yes		1.27	0.84, 1.92	0.2			
Study regions	950						
NCR		—	—				
ECR		1.29	0.97, 1.72	0.075			
^1^HR = Hazard ratio; CI = Confidence interval for simple Cox proporional hazard regression; ^2^HR = Hazard ratio, CI = Confidence interval for simple Cox proporional hazard regression							

Appendix 3 

Hyperparameter tuning process for the RSF model:

1. Randomized search for the RSF model

We performed a randomized search for hyperparameters to optimize the RSF model.

The hyperparameters considered in the search included the following:

a. Number of estimators (n_estimators)

b. Maximum depth of the trees (max_depth)

c. Maximum number of features considered for splitting (max_features)

d. Minimum samples required to split an internal node (min_samples_split)

e. Minimum samples required to be at a leaf node (min_samples_leaf)

2. RandomizedSearchCV

We utilized the RandomizedSearchCV function from scikit-learn to perform the randomized search.

The search was guided by a predefined search space, and it conducted multiple iterations to find the best combination of hyperparameters.

3. Out-of-bag (OOB) predictions

The hyperparameter tuning process for the RSF model was based on out-of-bag predictions within the training set.

This approach ensured that the validation data was not used for tuning, preventing any data leakage.

4. Cross-validation for other models

For other models, such as the support vector machine (SVM), we used k-fold cross-validation for hyperparameter tuning.

Similar to the RSF model, we ensured that the validation data was not included in the tuning process.

Appendix 4

The SVM model development and optimization process involved the following steps:

1. Data preprocessing

We initially copied the dataset and performed the necessary preprocessing steps on the features (X) and the target variable (y).

Categorical feature labels were encoded appropriately for SVM modeling.

2. Hyperparameter tuning with grid search

We conducted hyperparameter tuning using a grid search approach to find the best hyperparameters for the SVM model.

The hyperparameter of interest was "alpha," and we explored a range of values for alpha during the grid search.

3. Cross-validation for model development

The grid search for hyperparameter tuning was performed using k-fold cross-validation.

We utilized a ShuffleSplit cross-validation approach with five splits and a test size of 20% to ensure robust model development.

4. Model fitting and evaluation

After identifying the optimal hyperparameters, we fit the SVM model on the training data (X,y).

Model performance was evaluated using various metrics, including the concordance index, to assess the model's predictive accuracy.

5. Model testing

We applied the trained SVM model to the testing data (Xtest, ytest) to evaluate its performance on unseen data.

6. Receiver operating characteristic (ROC) analysis

We conducted ROC analysis to assess the SVM model's performance and generated ROC curves.

7. Time-dependent area under the curve (AUC) analysis

To evaluate the model's performance over time, we computed time-dependent AUC values and visualized them over different time intervals.

8. Boxplot visualization of hyperparameter tuning

We created boxplots to visualize the performance of the SVM model across different alpha values during hyperparameter tuning.

Appendix 5 

Categorization of High-Risk and Low-Risk Groups

To further gauge the discriminative power of our models, we employed the D-index, a metric that measures the degree of separation between patients in equally sized high-risk and low-risk groups. These categorizations were based on risk scores obtained from different models.

High-Risk and Low-Risk Groups

The process of categorizing patients into high-risk and low-risk groups involves assigning risk scores to each individual based on the model's predictions. Specifically, we calculate a risk score for each patient in the dataset, representing their estimated risk of experiencing the event of interest, which, in this study, is stroke mortality.

High-Risk Group

Patients with higher predicted risk scores are categorized into the high-risk group. These individuals are considered to have a higher likelihood of experiencing the event (stroke mortality) within a specific time frame.

Low-Risk Group

Conversely, patients with lower predicted risk scores are assigned to the low-risk group. These individuals are deemed to have a lower likelihood of experiencing the event within the same time frame.

Score Used for Differentiation

The risk scores used to differentiate between the high-risk and low-risk groups are derived directly from the models under evaluation. Each model produces a risk score for every patient in the dataset, reflecting the model's assessment of the individual's risk of stroke mortality.

For the Cox Proportional Hazard regression model, the risk scores are obtained as a continuous measure based on the Cox regression coefficients and the patient's feature values.

Similarly, for the support vector machine (SVM) and random survival forest (RSF) models, risk scores are generated as part of the model's prediction process.

The D-index is then calculated by assessing the extent of separation between these two groups based on their respective risk scores. A higher D-index value indicates a more significant distinction in predicted risks between the high-risk and low-risk groups, signifying a stronger discriminative ability of the model in stratifying patients based on their risk of stroke mortality.
